# Patient satisfaction with nurse-led telephone follow-up after curative treatment for breast cancer

**DOI:** 10.1186/1471-2407-10-174

**Published:** 2010-04-30

**Authors:** Merel L Kimman, Monique MF Bloebaum, Carmen D Dirksen, Ruud MA Houben, Philippe Lambin, Liesbeth J Boersma

**Affiliations:** 1 MAASTRO Clinic, Maastricht, the Netherlands; 2 Department of Radiation Oncology (MAASTRO), GROW - School for Oncology and Developmental Biology, Maastricht University Medical Centre, Maastricht, the Netherlands; 3 Department of Clinical Epidemiology and Medical Technology Assessment, Maastricht University Medical Centre, Maastricht, the Netherlands

## Abstract

**Background:**

Current frequent follow-up after treatment for breast cancer does not meet its intended aims, but does depend on expensive and scarce specialized knowledge for routine history taking and physical examinations. The study described in this paper compared patient satisfaction with a reduced follow-up strategy, i.e. nurse-led telephone follow-up, to satisfaction with traditional hospital follow-up.

**Methods:**

Patient satisfaction was assessed among patients (n = 299) who were participants of a randomized controlled trial investigating the cost-effectiveness of several follow-up strategies in the first year after treatment for breast cancer. Data on patient satisfaction were collected at baseline, three, six and 12 months after treatment, using the Dutch version of Ware's Patient Satisfaction Questionnaire III (PSQ III). In addition to general satisfaction, the PSQ III reports on satisfaction scores for technical competence, interpersonal aspects, and access of care. Regression analysis was used to predict satisfaction scores from whether or not nurse-led telephone follow-up was received.

**Results:**

Nurse-led telephone follow-up had no statistically significant influence on general patient satisfaction (p = 0.379), satisfaction with technical competence (p = 0.249), and satisfaction with interpersonal aspects (p = 0.662). Regarding access of care, patient satisfaction scores were significantly higher for patients receiving telephone follow-up (p = 0.015). However, a mean difference at 12 months of 3.1 points was judged to be not clinically relevant.

**Conclusions:**

No meaningful differences were found in satisfaction scores between nurse-led telephone and hospital follow-up in the first year after breast cancer treatment. With high satisfaction scores and the potential to substantially reduce clinic visits, nurse-led telephone follow-up may be an acceptable alternative to traditional hospital follow-up.

**Trial registration number:**

ISRCTN 74071417.

## Background

In most countries, follow-up after curative treatment for breast cancer consists of frequently scheduled follow-up visits (2-4 times a year) and an annual routine mammography [1]. The main objectives of these visits are the early detection of a locoregional recurrence or a second primary breast cancer, detection and registration of side effects of treatment, and provision of information and psychological support [1,2]. However, there is much debate whether these objectives are met in current clinical practice [3,4]. First of all, routine follow-up with clinical examination has been shown not to contribute to improved survival [5-9]. Additionally, outpatient clinic visits have been identified as a transition time of high stress, generating increased anxiety because of the risk of detecting a recurrence [10]. Also, some studies have suggested that outpatient clinic visits are insufficient to provide psychological support after breast cancer treatment, since these visits are often brief and do not include assessment of psychosocial problems [11]. Hence, current frequent follow-up visits seem to miss their most important goals, but do depend heavily on expensive and scarce specialized knowledge for routine history taking and physical examinations. As a result, the improvement in quality and efficiency of breast cancer follow-up care has been a government priority setting in many countries [12,13].

Alternative strategies for follow-up such as nurse-led and telephone follow-up have been proposed [14-16]. To improve healthcare, feedback from patients on these alternative models of follow-up is essential. Feedback evaluates the quality of the care provided, and can isolate problem areas and help generating ideas for further improvement [17,18]. Moreover, patients may respond better to treatment and comply better with instructions when they are satisfied with their care and treatment setting, improving their health outcomes [19-21].

Patient satisfaction with nurse-led follow-up and telephone services was found to be high [22,23], and good outcomes have been reported in terms of detecting medical problems and providing support to cancer patients [24,25]. Recent studies in breast cancer patients have also shown that telephone follow-up by specialist breast care nurses (BCNs) was well-received by patients [14,16]. A randomized clinical trial (RCT) by Beaver and colleagues, comparing nurse-led telephone follow-up with hospital follow-up showed that telephone follow-up significantly improved satisfaction, and it produced no excess anxiety compared with hospital follow-up [14]. A prospective cohort study by Montgomery and colleagues showed that an automated telephone system was easy to use and liked by most (71%) patients [16].

These studies provided positive results regarding patient satisfaction for follow-up after breast cancer for women on average one to two years after diagnosis. However, there is evidence that most physical and psychological recovery is achieved within the first year following treatment [26]. Therefore, the introduction of nurse-led telephone follow-up in the first year after treatment may affect patient satisfaction and quality of care differently than when applied in a later stage. It is expected that telephone follow-up by a BCN, who is familiar with the patient, can be appropriate to address psychological consequences after treatment [14]. Moreover, especially in the first year, telephone follow-up has the potential to reduce hospital visits.

This paper focused on patient satisfaction with nurse-led telephone follow-up compared to hospital follow-up in the first year after breast cancer treatment. Patient satisfaction was measured using a shortened (Dutch) version of the validated Patient Satisfaction Questionnaire (PSQ III) constructed by Ware and colleagues [27]. The PSQ III measures the multidimensional concept of patient satisfaction, capturing the most important characteristics of services and providers that might influence patient satisfaction with care. It is believed to reflect quality of care and patients' preferences [28].

## Methods

### Recruitment, design and sample

Patient satisfaction was assessed using data from patients who were participants of a randomized controlled trial (RCT) investigating the cost-effectiveness of nurse-led telephone follow-up after breast cancer (ISRCTN 74071417). A predefined (secondary) aim of the trial was to compare patient satisfaction between nurse-led telephone and hospital follow-up. Details of the trial design and protocol execution have been reported previously [29]. The study was a multicenter randomized trial, with a 2 × 2 factorial design. Between 2005 and 2008, 320 women were recruited through seven hospitals and two radiotherapy clinics in the South of the Netherlands. Participants were eligible for inclusion if they had completed breast cancer treatment with curative intent less than six weeks prior to randomization with a WHO performance score between 0-2, and were fluent in speaking and reading Dutch. Exclusion criteria were distant metastases, and/or participation in another clinical trial or medical illness requiring more frequent follow-up. All eligible patients received detailed study information, including information about the purpose and effectiveness of breast cancer follow-up and signs and symptoms of possible recurrences.

After written informed consent was obtained, participants were randomly assigned to one of four follow-up strategies (study arms) during the first 12 months after treatment; i.e. 1) hospital follow-up every three months, including mammography at 12 months; 2) nurse-led telephone follow-up every three months, plus hospital visit and mammography at 12 months; 3) arm 1 plus educational group program (EGP); 4) arm 2 plus EGP.

Randomization by minimization [30] was performed by the Comprehensive Cancer Center Limburg using a computerized randomization program (ALEA). Patients were pre-stratified by hospital and treatment modality (surgery, surgery + radiotherapy, surgery + chemotherapy, and surgery + radiotherapy + chemotherapy). The study protocol was approved by the Medical Ethical Review Board of MAASTRO Clinic (NL). All participating centers signed a local feasibility declaration, according to Dutch law and regulations, prior to inclusion of the first patient.

For the purpose of this analysis, hospital follow-up (arms 1 and 3) was compared to telephone follow-up (arms 2 and 4). In total, 162 patients were randomized to nurse-led telephone follow-up and 158 patients to traditional hospital follow-up.

### Procedures and intervention

In the Netherlands, follow-up after breast cancer in the first year after treatment consists of routine follow-up visits to the hospital (i.e. at three, six, nine and 12 months after treatment) [2]. A mammography is made at 12 months after the start of treatment, which is combined with a hospital follow-up visit. The provider of follow-up alternates between the surgeon, BCN, medical oncologist and radiation oncologist.

In patients randomized for hospital follow-up, follow-up was performed according to the above described Dutch guidelines. The follow-up visits consisted of physical examination and medical history and had a scheduled duration of approximately 10 minutes. In patients randomized for telephone follow-up, follow-up at three, six and nine months was performed by telephone, by a BCN or nurse practitioner (NP) working at this hospital, preferably the same nurse at each follow-up moment. At 12 months, a mammography was made and combined with a hospital follow-up. The telephone follow-up included a semi-structured interview in which physical -especially loco-regional- and psychosocial symptoms, treatment side effects and compliance with hormonal therapy were discussed. Furthermore, the BCN informed about general well-being of the patient, her family life, relationships, and work reintegration. Time scheduled for the telephone interview was approximately 15-20 minutes. If the patient had specific complaints or did not feel reassured, an additional appointment was made for her to come to the hospital. In order to adequately perform the telephone interview, all participating nurses attained four half-day training sessions, specifically developed for this study. In this training BCNs were informed on the most recent developments in breast cancer treatment and follow-up, and practiced their telephone communication skills with a simulation patient. Twenty-one BCNs from seven hospitals were trained.

### Outcome measures of effect

To assess patient satisfaction the Dutch version of the validated Ware's Patient Satisfaction Questionnaire III (PSQ III) was used (see Additional file 1). The PSQ III captures the most important characteristics of services and providers that might influence patient satisfaction with care. The Dutch version has left out financial aspects of the original questionnaire (i.e. questions 4, 10, 14, 19, 24, 27, 32 and 44), since in the Dutch health care system the personal financial situation is not directly related to the provision and quality of medical care. The structure and reliability of the PSQ III has been tested in a large sample of cancer patients in the Netherlands who were on average 8.6 months after treatment. Of this sample 31.1% were breast cancer patients. The PSQ III appeared an appropriate measure of cancer patients' satisfaction [27].

The PSQ III is a three factor model: besides general satisfaction (PSQ total), it consists of satisfaction with interpersonal aspects (IA) of the health care professional (i.e. providing explanations, listening skills, hasty behavior, empathy and respect), technical competence (TC) (i.e. knowledge of latest treatment techniques, competences of specialist/nurse) and satisfaction with access of care (AC) (i.e. easy and quick access to care, quality of care, waiting time). The questionnaire contains 43 favorably and unfavorably worded statements. Respondents are asked to indicate their agreement with the statements with respect to the care they received. The statements in the questionnaire all assume that medical care is provided by a doctor. To adjust to the study environment, the 'nurse' was added to the questionnaire. For example, 'the doctor/*nurse *who treats me has a genuine interest in me as a person' (question 17). Items are included in the questionnaire in random order and the answer alternatives range from 1 (strongly agree) to 5 (strongly disagree) [27]. Answers to favorably worded statements are reverse-coded, so that a higher number indicates more satisfaction. Sum scores were calculated for the PSQ total scale and for the three dimensions and subsequently transformed into a 100-point scale.

Possible response bias was investigated by using the matched-pairs method [31]. This methods checks whether a respondent tends to agree (or disagree) with two statements known to define opposite ends of the same satisfaction continuum, e.g. 'Doctors/nurses carefully listen to what I have to say' and 'Doctors/nurses sometimes ignore what I tell them'. The PSQ III includes five matched pairs. Each of these pairs is assigned a score of 0 if no response bias is present and 1 if response bias is present. Consequently, the theoretical range of response bias is 0 to 5; a score of 0 indicates no response bias, whereas a score of 2 or higher represents substantial bias.

The PSQ III was filled out before randomization at baseline, and three, six and 12 months after treatment. Patients received the questionnaire at home approximately one week after the follow-up visit or telephone interview and were asked to return it by mail in an enclosed envelope.

### Protocol compliance

Data on the actual follow-up received, thus number of hospital visits and telephone contacts with a BCN, were collected from patient files.

### Statistical analysis

The sample size for this study was determined by the sample size of the RCT, which was based on its primary outcome measure, i.e. health-related quality of life (HRQoL) at 12 months after end of treatment [29]. This paper deals with patient satisfaction at 12 months after treatment. We hypothesized that satisfaction with nurse-led telephone follow-up, regarding general satisfaction, interpersonal aspects, technical competences, and access of care, could be different from satisfaction with hospital follow-up. A difference in satisfaction scores of at least 0.5 standard deviation (SD), a medium effect, was considered to be clinically relevant. Assuming an SD of 17.9 for both groups [27], post hoc calculations showed that a sample of 299 patients from the RCT allowed to demonstrate a clinically relevant difference between nurse-led telephone and hospital follow-up (i.e. 8.95 points difference) with 90% statistical power and an α of 0.05.

Data were entered in a database by a professional center for data and information management and analyzed using SPSS version 17.0. Missing values within the survey's subscales were replaced using the regression function in SPSS if no more than half of the items were missing. If more than half of the items were missing the subscale was considered missing. If three or four PSQ III questionnaires were not returned, the patient was judged to be lost to follow-up. Missing data (i.e. subscales) from one or two questionnaires or missing covariates were imputed by means of Rubin's multiple imputation procedure [32,33].

Differences in patient characteristics between telephone and hospital follow-up were compared using the X^2 ^test and independent sample t-test. Regression analyses were used to predict outcome differences by including or excluding the intervention. Linear mixed models were fitted with telephone follow-up (yes/no), as a fixed factor, and patient as random factor. In addition, time since end of treatment, age, hospital, treatment modality, education level, and the outcome variable at baseline were brought into the model. In the primary analysis data were analyzed according to the intention to treat principle. However, since protocol violation may bias the results (in either direction), per protocol analyses including only patients who properly followed the study protocol were also performed and reported [34,35].

## Results

### Patients

Data from 299 patients were available for the purpose of this analysis. In the RCT, 21 of 320 randomized patients had dropped out of the trial due to various reasons (e.g. development of metastases, recurrence, or three or more missing questionnaires). Data of 149 patients randomized to hospital follow-up and data of 150 patients randomized to telephone follow-up were available for the evaluation of patient satisfaction. Mean sample age was 56 years (SD = 9.9). Sociodemographic and treatment characteristics as well as baseline satisfaction scores were similar in the two groups (table 1).

**Table 1:** Sociodemographic and treatment characteristics of participants (n = 299) according to hospital or telephone follow-up.

	Total group (n = 299)	Hospital follow-up (n = 149)	Telephone follow-up (n = 150)	p-value
**Age at randomization (years)**				
Mean (SD)	56 (9.9)	56 (10.7)	55 (9.0)	0.50
Range	23-78	23-78	34-75	0.06
< 45	36 (12)	18 (12)	18 (12)	
45-64	203 (68)	93 (62)	110 (73)	
≥ 65	60 (20)	38 (26)	22 (15)	
				
**Level of education**				0.36
Low	102 (34)	45 (30)	57 (39)	
Middle	118 (40)	62(42)	56 (37)	
High	79 (26)	42 (28)	37 (24)	
				
**Marital status**				0.15
Married	212 (71)	109 (73)	103 (69)	
Unmarried	33 (11)	16 (11)	17 (11)	
Cohabiting	29 (10)	9 (6)	20 (13)	
Widowed	25 (8)	15 (10)	10 (7)	
				
**Treatment modality**				0.99
Surgery	29 (10)	15 (10)	14 (9)	
Surgery + radiotherapy (RT)	178(60)	89 (60)	89 (60)	
Surgery + chemotherapy (CH)	15 (5)	7 (5)	8 (5)	
Surgery + RT + CH	77 (25)	38 (25)	39 (26)	
				
**Hormonal therapy**				0.59
Yes	94 (31)	50 (34)	44 (29)	
No	205 (69)	99 (66)	106 (71)	
				
**Baseline satisfaction scores (mean (SD)**				
General satisfaction	77.2 (19.5)	77.7 (14.2)	76.7 (18.9)	0.65
Interpersonal aspects	81.8 (17.5)	81.4 (17.6)	82.2 (17.5)	0.70
Access of care	76.9 (14.0)	77.4 (14.2)	76.4 (13.8)	0.54
Technical competence	77.0 (16.3)	76.7 (17.2)	77.4 (15.4)	0.69

Values are numbers (percentages) unless stated otherwise.

### Compliance to the protocol

Ten patients randomized for telephone follow-up preferred to receive hospital follow-up instead, and case record forms indicated that 20 patients with telephone follow-up received only one telephone follow-up contact, which was considered as protocol violation. Hence, 120 of the 150 patients in the telephone follow-up group, received telephone follow-up according to the protocol. Since hospital follow-up represented usual care, no protocol violations were apparent in this group.

The 30 patients who violated the protocol in the telephone group did not differ from other patients in this group regarding age, education, treatment modality and satisfaction scores at baseline (all p-values > 0.05).

Table 2 shows the mean number of total hospital visits and telephone contacts with the breast care nurse per group for the study period of one year. In the hospital group patients had on average 5.9 visits to the hospital, of which four visits were conform protocol and 1.9 were additional visits. In the telephone group patients had on average 2.4 telephone contacts with the BCN and 3.4 visits to the hospital, of which one hospital visit was conform protocol and 2.4 were additional visits.

**Table 2:** Number of contacts with medical specialist (MS) and breast care nurse (BCN) according to follow-up group (hospital and telephone) in one year.

	Hospital follow-up			Telephone follow-up		
	
	Conform protocol	Additional contacts	Total contacts	Conform protocol	Additional contacts	Total contacts
**Intention to treat analysis**						
Visits hospital (MS or BCN)	4	1.9	5.9 (2.2)	1	2.4	3.4 (2.4)
Telephone contact BCN	0	0.1	0.1 (0.4)	3	-0.6^a^	2.4 (1.1)
						
**Per protocol analysis**						
Visits hospital (MS or BCN)	4	2.9	5.9 (2.2)	1	2.0	3.0 (2.3)
Telephone contact BCN	0	0.1	0.1 (0.4)	3	-0.2^a^	2.8 (0.7)

Numbers are means and standard deviations.

^a ^Negative numbers imply fewer contacts than set by the protocol

### Patient satisfaction

Since patients who violated the protocol for telephone follow-up did not differ from patients who adhered to telephone follow-up, results from the intention to treat analyses are discussed as the primary outcomes in this section.

**Figure 1:**
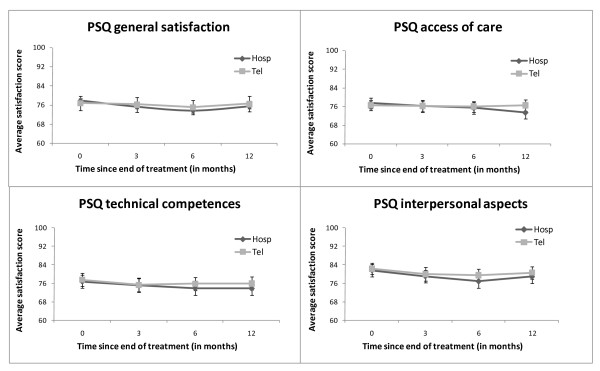
**Average satisfaction scores for hospital follow-up compared to nurse-led telephone follow-up**. Figure 1 presents average satisfaction scores for general satisfaction, access of care, technical competences and interpersonal aspects, at baseline, three, six and 12 months after treatment, for hospital follow-up compared to nurse-led telephone follow-up. Error bars represent 95% confidence intervals.

In the first year after treatment patient satisfaction scores were high in both groups in all subscales of the PSQ III, at all time points (see figure 1). Table 3 shows satisfaction scores at 12 months after treatment. General patient satisfaction at 12 months was 75.3 (SD = 19.6) in the hospital follow-up group and 76.4 (SD = 19.7) in the telephone follow-up group. Patient satisfaction regarding technical competence was 73.7 (SD = 17.9) for the hospital follow-up group and 75.8 (SD = 16.8) for the telephone follow-up group. Satisfaction with interpersonal aspects was 78.7 (SD = 18.5) for the hospital follow-up group and 78.9 (SD = 17.6) for the telephone group. Regarding access of care satisfaction for the hospital follow-up group was 73.3 (SD = 15.7) and for the telephone follow-up group 76.4 (SD = 15.6). Regression analysis showed that nurse-led telephone follow-up had no statistically significant influence on general patient satisfaction (p = 0.379), satisfaction with technical competence (p = 0.249), and satisfaction with interpersonal aspects (p = 0.662). Regarding access of care, patient satisfaction was significantly higher for patients receiving telephone follow-up (p = 0.015). However, a mean difference at 12 months of 3.1 points may be statistically significant, but was not considered clinically relevant (i.e. < medium effect).

**Table 3:** Outcome findings by study group adjusted for treatment, hospital, outcome variable at baseline, age, educational level and time since treatment.

	Mean (SD) telephone follow-up at 12 months	Mean (SD) hospital follow-up at 12 months	Estimated difference	95% Confidence Interval for difference^a^	p-value
**Intention to treat analysis**	(n = 150)	(n = 149)			
					
General satisfaction					
Telephone vs. hospital f-up	76.4 (19.7)	75.3 (19.6)	1.86	-2.30 to 6.03	0.379
Interpersonal aspects					
Telephone vs. hospital f-up	80.5 (17.6)	78.7 (18.5)	0.91	-3.18 to 5.00	0.662
Access of care					
Telephone vs. hospital f-up	76.4 (15.6)	73.3 (15.7)	3.10	.71 to 6.70	0.015
Technical competence					
Telephone vs. hospital f-up	75.8 (16.8)	73.7 (17.9)	2.13	-1.51 to 5.77	0.249
					
**Per protocol analysis**	(n = 120)	(n = 159)			
					
General satisfaction					
Telephone vs. hospital f-up	76.2 (19.7)	75.7 (19.9)	1.13	-3.14 to 5.39	0.604
Interpersonal aspects					
Telephone vs. hospital f-up	81.3 (17.5)	79.2 (18.2)	0.55	-3.63 to 4.73	0.796
Access of care					
Telephone vs. hospital f-up	75.8 (16.1)	74.7 (15.3)	3.10	-0.13 to 6.32	0.060
Technical competence					
Telephone vs. hospital f-up	75.1 (16.7)	74.1 (18.0)	1.76	-1.91 to 5.42	0.347

**^a ^**Positive differences imply a higher level of satisfaction in the telephone group.

In the per protocol analyses, ten patients who had refused telephone follow-up after randomization were analyzed in the hospital follow-up group, while the 20 patients who had not properly received telephone follow-up were excluded from the analyses. Per protocol analyses showed almost identical results to intention to treat analyses (table 3). However, in contrast to the intention to treat analysis, higher patient satisfaction scores in the telephone group regarding access of care were not significant (p = 0.060).

### Response bias

Response bias in the questionnaire was tested on the 12 months data. We found neglectable response bias scores for the tendency to disagree regardless of content (0.7% of respondents), but 14% of respondents showed substantial response bias, i.e. agreed with two or more (out of five) opposite statements regardless of their content. Nevertheless, an analysis with a calibrated sample of respondents without response bias (n = 257) showed identical results to the main analysis regarding all factors of satisfaction (data not shown).

## Discussion

To improve follow-up care after breast cancer, feedback from patients on satisfaction is important since it provides information on the quality of care received. Furthermore, patients may comply better with a specific follow-up strategy when they are satisfied with their care and follow-up setting. Insight into patient satisfaction when evaluating telephone follow-up was therefore an important aspect of our RCT.

The results of this study show that nurse-led telephone follow-up after breast cancer may well be an appropriate alternative to hospital follow-up. Patient satisfaction scores at 12 months after treatment were high in all subscales of the PSQ III for both nurse-led telephone and hospital follow-up. No meaningful differences were found between the two types of follow-up in scores for general satisfaction, satisfaction with interpersonal aspects, satisfaction with technical competences of staff and access of care. Since equivalence was seen as a positive outcome in this study, it was important to carefully analyze protocol violators and perform both intention to treat and per protocol analyses [35]. Both types of analyses showed almost identical results. This was expected since the 30 patients who violated the protocol in the telephone group did not significantly differ from other participants in this group regarding age, level of education, treatment received and baseline satisfaction scores.

To our knowledge, this was the first study to evaluate the multidimensional concept of patient satisfaction with nurse-led telephone follow-up specifically aimed at the first year after breast cancer treatment. It is also one of the few studies that used a validated questionnaire to measure patient satisfaction [36]. Although the PSQ III was not specifically developed to measure satisfaction with follow-up care and response bias is a documented problem of the questionnaire, the Dutch version was validated among breast cancer patients after treatment and satisfaction and response bias scores found in our study were similar to the norm scores found by Hagedoorn and colleagues [27]. Furthermore, the pragmatic nature of the RCT led to the inclusion of a broad range of patients regarding age, treatment and location of treatment, including a sample representative of breast cancer patients in the Netherlands [37].

In general, the positive findings in this study were similar to findings reported in other studies investigating nurse-led telephone follow-up [14,16,27,38]. Even though the results are difficult to compare, there is a strong trend towards acceptability, feasibility and good patient satisfaction scores for telephone follow-up. The recent study by Beaver and colleagues is most comparable to our study. In their randomized clinical trial (n = 374), a structured telephone intervention was developed and nurses received four half day training sessions, similar to our study. Patient satisfaction was evaluated at the beginning, middle, and end of the trial, by asking participants if they were satisfied with information received and whether the appointment had been helpful in dealing with their concerns. It was found that patient satisfaction was significantly higher for telephone follow-up compared to hospital follow-up at the middle and at the end of the trial. Women were recruited between 0.5 and 106 months after the end of treatment and remained in the trial for a mean of 24 months [14]. In our study women entered immediately after treatment and remained in the trial for 12 months. Thus, the study by Beaver and colleagues and our study can be seen as complementary; both provide evidence that telephone follow-up can be appropriate for patients in different phases after breast cancer treatment [14].

Several explanations can be hypothesized for the high satisfaction scores with nurse-led telephone follow-up. First of all, telephone follow-up was performed by a specialized breast care nurse, most often a nurse familiar to the patient from the time of diagnosis and treatment. It was expected that patients felt comfortable expressing emotions and concerns to this nurse [11]. The breast care nurses were also specifically trained to meet information and psychological needs. The follow-up may take up to 20 minutes and was done by open discussion, offering the patient the opportunity to discuss issues they were most concerned with. Furthermore, nurse-led telephone follow-up provided continuity to the patient. In general, the same nurse provided the telephone follow-up for a patient, which is different from hospital follow-up where patients were seen by the medical oncologist, radiation oncologist, surgeon, or breast care nurse.

Despite positive results, the conclusion that nurse-led telephone follow-up provides equal satisfaction compared to hospital follow-up must be made carefully, taking into account several possible limitations of this study. First, of eligible patients 64% declined participation in the randomized trial. This might be due to a lack of knowledge about the purpose and effectiveness of follow-up [39,40]. Moreover, the fact that patients were informed about the usual care (hospital follow-up) before participation may have also negatively influenced the participation rate, since patients generally have a preference for what they know best, the so-called 'status quo bias' [41]. Hence, patient education on follow-up will need special attention in future trials or when implementing telephone follow-up. The relatively low participation rate of the RCT may have an impact on the generalizabilty of our results. It specifically influenced this patient satisfaction study, since patients who chose to participate in the RCT may have had either no strong preference for a follow-up strategy, or positive expectations of the interventions. Thus, patients may have been somewhat uncritical of the care provided, or prior expectations about follow-up may have influenced expressed satisfaction [42,43]. In other words, patients may have expressed satisfaction no matter what care was provided. It is unclear whether and if so, to what extent, the sample selection has influenced our results. Additionally, patients who had developed a recurrence or metastatic disease in the study period were lost to follow-up. Hence, the analysis included a sample of patients who remained disease-free in the first year after treatment. It may be speculated that these patients will generally show high satisfaction scores.

Second, one in five patients randomized to telephone follow-up did not receive telephone follow-up according to protocol, which may in itself be seen as evidence that the two follow-up strategies were not entirely equal in terms of preference and feasibility. Indeed, ten patients requested to receive hospital follow-up directly after having been randomized for telephone follow-up. However, most other protocol violations were related to logistic difficulties or health-related problems (e.g. the patient needed to visit the hospital for a complaint, but was unintentionally not re-entered in the telephone follow-up). Moreover, even though hospital follow-up was better adhered to in the trial and generally preferred beforehand, per protocol analyses showed high satisfaction scores for telephone follow-up, equal to patients who followed hospital follow-up.

Third, it must be recognized that all patients received some follow-up in the hospital, also the patients randomized to telephone follow-up, which may have contributed to the fact that no differences between the two forms of follow-up were found.

Finally, other outcomes besides patient satisfaction are relevant when exploring alternative follow-up strategies for breast cancer, such as the effectiveness in terms of health-related quality of life, emotional functioning, feelings of anxiety and costs. These outcome measures will be assessed before implementation of telephone follow-up (paper in preparation).

## Conclusion

This study showed that regular telephone contact with a breast care nurse and a one-year mammography combined with a hospital visit was equal to traditional hospital follow-up visits, in terms of general satisfaction, and satisfaction with regard to technical competences of staff, access of care and interpersonal aspects. It is concluded that nurse-led telephone follow-up in the first year after breast cancer treatment may be an appropriate and acceptable alternative to hospital follow-up.

## Competing interests

The authors declare that they have no competing interests.

## Authors' contributions

MK and MB were responsible for the data collection, analysis and drafting of the manuscript. RH provided statistical assistance. RH, LB, CD and PL all participated in discussing the design of this study and development of the research protocol for the RCT. All authors read and corrected draft versions of the manuscript.

## Pre-publication history

The pre-publication history for this paper can be accessed here:

http://www.biomedcentral.com/1471-2407/10/174/prepub

## Supplementary Material

Additional file 1 **PSQ III questionnaire**. Patient Satisfaction Questionnaire. Click here for file
