# Supplementary figures and images for: Patient satisfaction with nurse-led telephone follow-up after curative treatment for breast cancer
Source: BMC Cancer. 2010 Apr 30;10:174. doi: 10.1186/1471-2407-10-174 (PMC2880988; doi:10.1186/1471-2407-10-174)

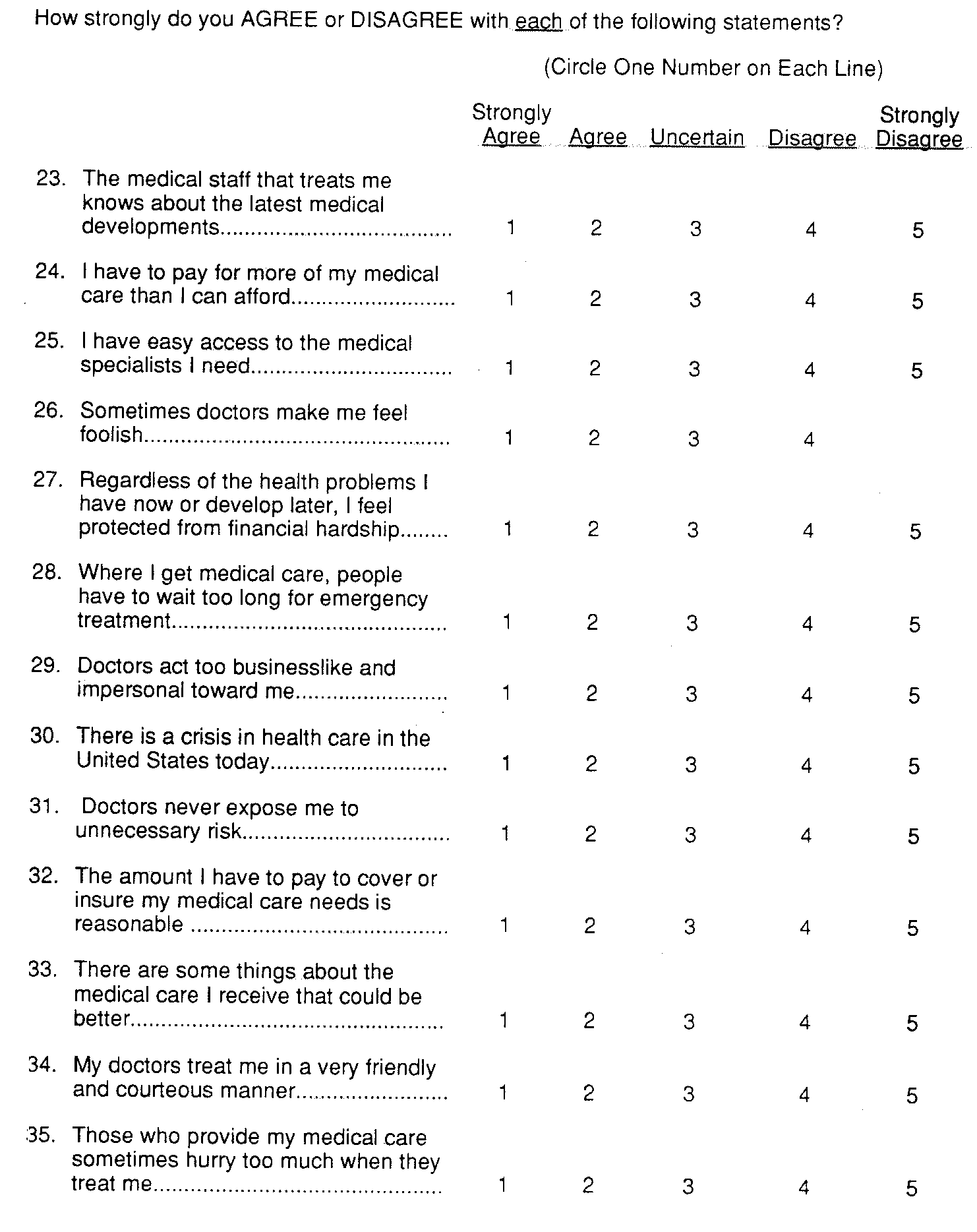


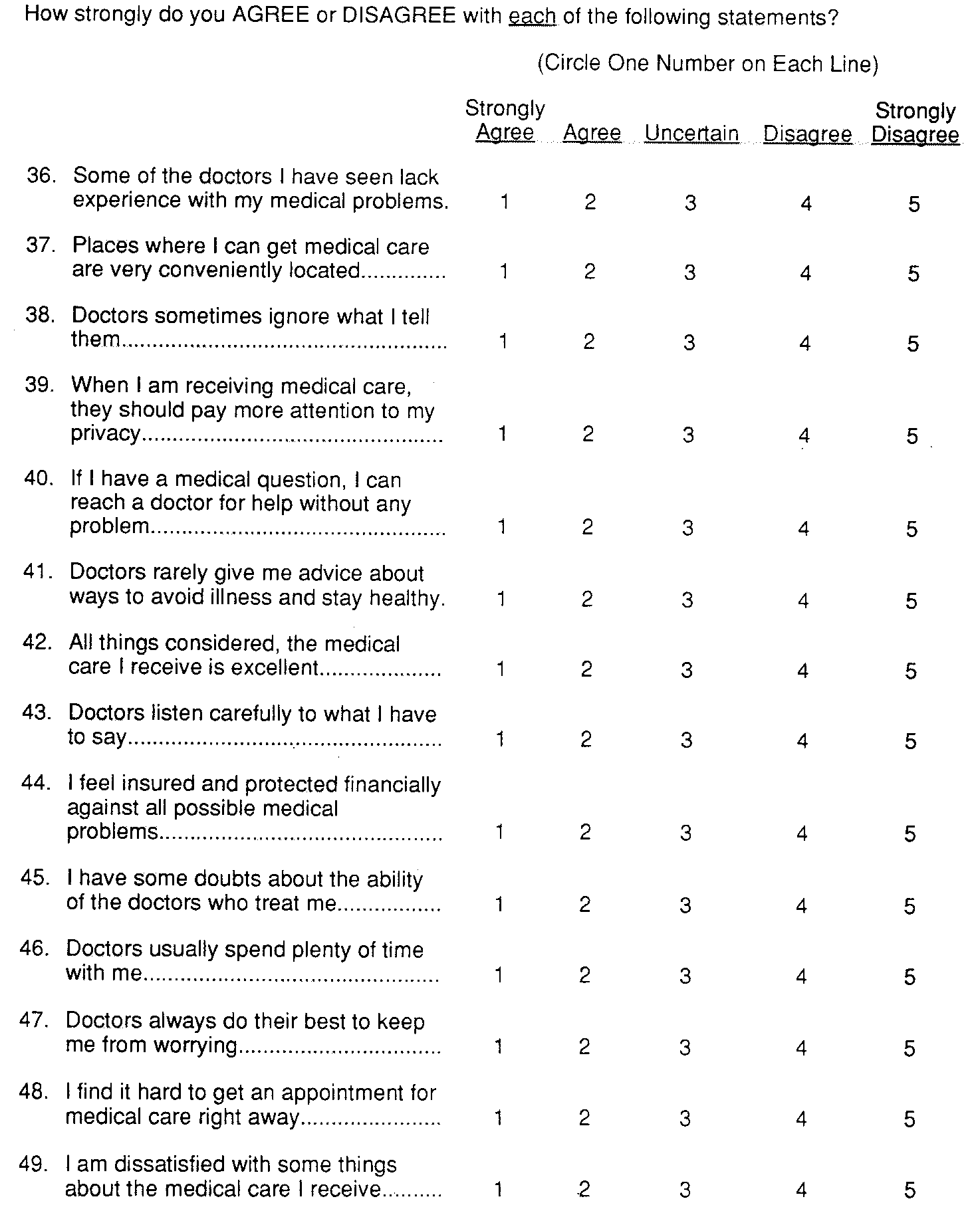

Supplement: Additional file 1 — PSQ III questionnaire. Patient Satisfaction Questionnaire. [file 1471-2407-10-174-S1.DOC]
